# The validity of the EQ-5D-3L items: an investigation with type 2 diabetes patients from six European countries

**DOI:** 10.1186/s12955-014-0181-5

**Published:** 2014-12-05

**Authors:** Uwe Konerding, Sylvia G Elkhuizen, Raquel Faubel, Paul Forte, Tomi Malmström, Elpida Pavi, MF Bas Janssen

**Affiliations:** Trimberg Research Academy, University of Bamberg, Bamberg, D-96045 Germany; Institute of Health Policy & Management, Erasmus University Rotterdam, PO Box 1738, Rotterdam, DR 3000 The Netherlands; Joint Research Unit in Biomedical Engineering, IIS La Fe- Universitat Politècnica de València, Bulevar sur s/n, 46026 Valencia, Spain; The Balance of Care Group, 39a Cleveland Road, London, N1 3ES UK; Department of Industrial Engineering and Management, Aalto University, PO Box 15500, Espoo, Aalto 00076 Finland; Department of Health Economics, National School of Public Health, 196 Alexandras Ave, 115 21 Athens, Greece; Department of Psychiatry, Section Medical Psychology & Psychotherapy, Erasmus MC, PO Box 2040, CA 3000 Rotterdam, The Netherlands

**Keywords:** Health-related quality of life, EQ-5D-3L, Validity, Inter-country, Methodology

## Abstract

**Background:**

Most previous studies concerning the validity of the EQ-5D-3L items refer to applications of only a single language version of the EQ-5D-3L in only one country. Therefore, there is little information concerning the extent to which the results can be generalised across different language versions and/or different countries. Here the validity of the EQ-5D-3L items is investigated for six different language versions in six different countries.

**Methods:**

Data came from 1341 type 2 diabetes patients (England: 289; Finland: 177; Germany: 255; Greece: 165; the Netherlands: 354; Spain: 101). The relationships of the five EQ-5D-3L items with seven different test variables (age, gender, education, previous stroke, problems with heart, problems with lower extremities, problems with eyes), were analysed for each combination of item and test variable. For each combination two logistic regression models with the dichotomised EQ-5D-3L item as dependent variable were computed. The first model contained the test variable and dummy coded countries as independent variables, the second model additionally the terms for the interaction between country and test variable. Statistically significant better fit of the second model was taken as evidence for country specific differences regarding the relationship. When such differences could be attributed mainly to one country the analyses were repeated without the data from this country. Validity was investigated with the remaining data using results of the first models.

**Results:**

Due to lack of variation in the Spanish data only 31 of the originally intended 35 interaction tests could be performed. Only three of these yielded a significant result. In all three cases the Spanish data deviated most. Without the Spanish data only 1 of the 35 interaction tests yielded a significant result. With 3 exceptions, the tendency of reporting problems increased with age, female gender, lower education, previous stroke, heart problems, problems with lower extremities and problems with eyes for all EQ-5D-3L items.

**Conclusion:**

The results concerning the European Spanish version are ambiguous. However, the items of the English, Finnish, German, Greek and Dutch versions of the EQ-5D-3L relate in substantially the same way to the test variables. Mostly, these relationships indicate the items’ validity.

The EQ-5D-3L with its 153 different official language versions and a further 19 language versions awaiting approval is currently the most widely used preference-based instrument for measuring health related quality of life [1-4]. The EQ-5D-3L consists of (1) a descriptive system comprising five questionnaire items and (2) a visual analogue scale. The questionnaire items address five different dimensions of health: (1) mobility; (2) self-care; (3) usual activities; (4) pain/discomfort; and (5) anxiety/depression. Three answer categories are provided for each item with the first category referring to the best state. The EQ-5D-3L can be applied for two purposes: describing health states; and constructing indices which represent the utilities of these health states. Several scoring functions for assigning utilities to health states are available and most of these refer to one specific language version of the EQ-5D-3L [5].

Whatever purpose the EQ-5D-3L items are used for, they need to be valid. This means, in the first instance, that they should adequately describe the respondents’ actual health states. This can best be investigated by analysing the relationships between the items and some more concrete indicators of actual health. There are several studies which refer to these relationships [6-17] and which, together, constitute a remarkable evidence basis for judging the validity of the EQ-5D-3L items. For a deeper understanding, however, it is also interesting to know how the items relate to socio-demographic variables such as age, gender and education. As far as these socio-demographic variables are related to actual health, the relationships of these variables with the EQ-5D-3L items also provide information about the items’ validity. There are several studies in which these relationships have been investigated [6,11,14,15,17-20] and the findings from these studies enrich the empirical knowledge relevant for judging the validity of the EQ-5D-3L items.

However, most of the studies just mentioned have been performed in only one specific country using only one specific language version of the EQ-5D-3L. There are only four studies in which different language versions have been applied. Three of these have been performed using different language versions within the same country [12,15,18] and only one study referred to data from different countries [19]. Analyses which are relevant for investigating validity and which have been performed separately for either the different language versions or the different countries respectively have been reported for only two of the four studies [12,19]. Hence, there is not much empirical evidence concerning the extent to which results referring to the EQ-5D-3L items can be generalised across different language versions and/or across different countries. In fact, the results of one of the few relevant studies [12] even suggest that this is not the case. Obtaining more information about the generalizability of findings concerning the EQ-5D-3L items would require more studies in which different language versions of the EQ-5D-3L are applied in different countries.

A study with the features just mentioned is presented here, performed on patients with type 2 diabetes. Type 2 diabetes is a chronic insufficiency in the processing of glucose in the blood caused by too little insulin being produced by the body or by the available insulin working ineffectively. In the long run diabetes can lead to severe complications; particularly stroke, heart attack, kidney failure, ulcers of the lower extremities and impairment of sight [21,22].

Patients from England, Finland, Germany, Greece, the Netherlands and Spain were investigated and the corresponding EQ-5D-3L language versions were used. The data from this study are analysed with regard to two objectives:

1. to investigate the generalizability of findings concerning the EQ-5D-3L items across the study countries, and
2. to investigate the validity of the EQ-5D-3L items on the basis of the data from those countries for which the findings concerning the EQ-5D-3L items are substantially the same.

## Methods

### Study participants and study conduction

The analyses were performed using data from surveys of type 2 diabetes patients in England, Finland, Germany, Greece, the Netherlands and Spain which were conducted during a major European project concerned with health provider networks [23]. Inclusion criteria for participants were 1) that they were being treated for type 2 diabetes by the health providers investigated in the project and 2) that they were at least 18 years old. The health providers investigated in the study selected the patients to be approached for participation according to criteria defined by the researchers. The patients were then contacted either by post or by being directly given the questionnaire when visiting their health care provider. The patients who participated in the survey completed their questionnaires on their own without personnel from the service provider or a researcher beside them. Depending on what was the most feasible method for the provider the participants returned their completed questionnaires either by mail directly to the local project study centres, or to the care provider who then passed them on to the study centres. All surveys had been approved by national ethical committees and took place between October 2011 and March 2012.

### Questionnaire

The questionnaire used for the surveys contained items addressing socio-demographic features, health, health-related behaviour and health treatment. Most of these items were not included in the analyses presented here. The questionnaire items included were those concerning age, gender, educational attainment, and secondary complications of diabetes, and all EQ-5D-3L items. There were also two questions concerning the participant’s competence in mastering the questionnaire language. In the English version of the questionnaire the first question was ‘What is your first language?’ and the categories ‘English’ and ‘Other, please specify’ were given as answer options. The second question was ‘If English is not your first language, how well do you master it?’ with the answer options ‘Not at all’, ‘Poorly’, ‘Moderately’, ‘Well’ and ‘Perfectly’. In the other language versions the word ‘English’ was replaced with the word for the language in which the questionnaire was formulated. Both questions were relevant in selecting which questionnaires were to be included for the analyses.

Educational attainment was assessed by asking participants whether they had left school after the minimum school leaving age of their country. Those answering ‘yes’ were classified as having a lower level of educational attainment than those who answered ‘no’. After lengthy discussions with researchers from all six investigated countries this assessment turned out to fit best to all the six corresponding education systems which are all diverse in many respects.

Five secondary complications of type 2 diabetes were considered in the questionnaire: stroke, kidney failure, heart problems, problems concerned with the lower extremities and problems with eyes. Each complication was addressed by a single question. Stroke was assessed by asking if they had ever had a stroke, and kidney failure by asking if they were dependent on dialyses or had had a previous kidney transplant. The other three secondary complications were addressed by questions each of which in turn had three answer options. For heart problems these were: no problems; having had a by-pass or suffering from angina, but never having had a heart attack; and having had a heart attack. For problems with lower extremities the options were: no problems with legs, feet and toes caused by diabetes; having lesions in legs, feet or toes caused by diabetes, but no amputation caused by diabetes; and amputation of a toe, foot or leg due to problems caused by diabetes. The three answer options for eye problems were: no problems; problems caused by diabetes, but no blindness; and blindness as a result of diabetes.

### Statistical analyses

Questionnaires were excluded from analyses if the participant could not sufficiently master the questionnaire language and/or if there were too many missing values for the variables under consideration. Sufficient mastering of the questionnaire language was defined as either being a native speaker or as mastering the questionnaire language at least ‘well’. Having too many missing values was defined as there being either more than one missing value for the three socio-demographic questions, or more than one missing value for the five questions concerning the secondary complications or more than one missing value for the five EQ-5D-3L items. The latter criterion was a compromise between the demands of not relying on information that was too questionable and of not excluding too many participants. If there was no more than one missing value for each of the three question categories, this could still be attributed to casual slips by the respondent. More missing values than this were taken as an indicator of fundamental insufficiencies in the respondent's answers.

To obtain a basic understanding of the data, the distributions of the three socio-demographic features and the five secondary complications were analysed for each country separately, and for all countries together. Age was treated both as a continuous variable and as a categorical variable with the categories ’18-54’, ’55-64’, ’65-74’, and ‘older than 74’. These categories were chosen because they were expected to cover qualitatively different age ranges. Country differences with regard to age were tested by computing a multivariate linear regression with age as the dependent variable and dummy coded countries as independent variables. Country differences with regard to the remaining socio-demographic variables, stroke, and kidney failure were tested using Pearson’s chi-square test. Country differences on the three ‘three-categorical’ secondary complications (problems with heart, lower extremities, or eye problems) and the five EQ-5D-3L items were analysed using the Kruskal-Wallis test. This test is specially designed for comparing values of variables with an ordinal scale level. It was chosen because the three-categorical items for the secondary complications as well as the EQ-5D-3L items possess only this scale level. Bivariate relationships of the socio-demographic features and the secondary complications with the EQ-5D-3L items were also investigated using Kruskal-Wallis tests. The remaining analyses directly refer to the two objectives presented in the introduction.

#### Generalizability of findings concerning the EQ-5D-3L items

As mentioned above the validity of the EQ-5D-3L items is usually investigated by analysing the relationships of these items with demographic features and with more objective features of health. Therefore, the cross-national generalizability of findings concerning the EQ-5D-3L items was investigated here with regard to exactly these relationships. To simplify these analyses the five EQ-5D-3L items and all three-categorical items referring to secondary complications were dichotomised with one category being ‘no problems at all’ and the other ‘any problems’. The secondary complications which were measured with three-categorical items were problems with heart, lower extremities and eyes. The items referring to stroke and to kidney failure were already binary variables. As country specific differences regarding the relationships between the EQ-5D-3L items and other variables can only be reasonably investigated when there are values for all levels of the investigated variables in all countries those socio-demographic features and secondary complications were excluded from the analyses which did not meet this criterion. The included variables will be referred to as the ‘test variables’ below.

To test whether there are country-specific differences regarding relationships between the EQ-5D-3L items and the test variables two logistic regression models were compared for each combination of EQ-5D-3L item and test variable. Both models contained the respective dichotomised EQ-5D-3L item as dependent variable. The first of the two models contained the test variable and dummy coded country as independent variable; the second model contained additionally terms for the interaction between countries and test variable. Statistically significant better fit of the second model was taken as evidence for country specific differences regarding the relationship. This was tested using a likelihood ratio test. To get also descriptive measures for the improvement of model fit Nagelkerke’s pseudo R-square was computed for each model. Nagelkerke’s pseudo R-square reflects how all included independent variables together explain the dependent variable. This statistic is zero when the independent variables do not explain the dependent variable at all. When, in contrast, the independent variables explain the dependent variable completely this statistic is equal to one [24].

For those combinations of EQ-5D-3L items and test variable for which the interaction terms led to a statistically significant improvement of the model fit further analyses were performed. These analyses aimed to identify that country for which the investigated relationship differed most from the other countries. For this purpose, logistic regression models with the dichotomised EQ-5D-3L item as dependent variable were computed for each country separately. These country specific regression models contained only the test variable as independent variable. This was always one dummy variable (except for age, which was modelled with three dummy variables). It was determined how the country specific regression coefficients for these dummy variables deviated from the corresponding regression coefficients in the models which were computed for all countries together without using interaction terms. These deviations directly reflect how much the relationship for the respective country differs from the common core of all countries. When it was mainly the same country which deviated most from the common core when there were statistically significant interactions then all analyses just described were repeated without the data from this country.

#### Validity of the EQ-5D-3L items

The validity of the EQ-5D-3L items was investigated with the data from those countries for which there were no essential interaction effects. This was achieved through interpreting the results from those regression models which were computed for all countries together and where interaction terms as independent variables were excluded.

## Results and discussion

More than 6000 questionnaires were distributed of which 1638 were returned and 1341 met the inclusion criteria (see Table 1). The proportion of excluded questionnaires was largest in England (39.2%) which was due to the fact that about 40% of all respondents in this sample were of Bangladeshi ethnicity who, due to lower levels of stated proficiency in the English language, did not meet the inclusion criteria for this analysis. Altogether, 21.6% of the distributed questionnaires were finally included in the analyses and these proportions vary from 8.6% for England to 55.2% for Germany. As a consequence percentages and means determined from these data might deviate from those means and percentages which would have been obtained for the total sample. However, relationships between variables can rather be expected to be the same for responders and non-responders. Hence, the low response rates will most probably not constitute a too great danger for the validity of the analyses concerning the relationships between the EQ-5D-3L items and the test variables.

**Table 1:** **General information about the sample**

	**Questionnaires distributed**	**Questionnaires returned**	**Sufficient language competence**	**Sufficient data** ^**a**^	**Participants included**
England	3343	475	313	436	289
Finland	436	183	183	177	177
Germany	462	286	282	259	255
Greece	600	179	179	165	165
The Netherlands	779	400	387	364	354
Spain	625	115	115	101	101
All countries	6245	1638	1459	1502	1341

^a^Participants who answered at least two of the three socio-demographic questions, at least four of the five questions concerning secondary complications, and at least four of the five EQ-5D-3L questions.

The mean age of all included participants was 65.4 years. The majority of the participants was male (56.9%), had a higher education attainment (53.6%), and no secondary complications (ranging from 78.3% for no heart complications to 99.7% for no kidney failure) (see Table 2). For the three socio-demographic variables country differences were statistically significant except for gender. For the five secondary complications country differences were significant except for kidney failure (see Table 2). As there were three countries in which there were no participants with kidney failure, this variable could not be applied for the analyses intended here. For all four secondary complications with statistically significant country differences, the best health states were reported in the Netherlands. Data were available for all categories of the five EQ-5D-3L items; however only for ‘usual activities’ , ‘pain/discomfort’ , and ‘anxiety/depression’ were there data for all categories in every country (see Table 2). There were significant differences between the countries for all five items. The Dutch participants had the lowest rates of reporting problems for mobility (31.3%), self-care (5.2%), usual activities (22.7%) and pain and/or discomfort (43.2%) and the Finnish participants had the lowest rate of reporting problems with depression and/or anxiety (11.4%).

**Table 2:** **Distributions of the investigated variables** ^**a**^

	**England**	**Finland**	**Germany**	**Greece**	**Nether-lands**	**Spain**	**All countries**
Socio-demographic features							
Age (p < 0.001)^b^							
Mean	63.2	64.2	66.1	66.4	65.9	68.5	65.4
SD	12.8	9.5	11.4	10.7	10.3	11.6	11.2
Minimum	28	34	21	30	38	30	21
Maximum	90	98	92	89	89	92	98
<55	69 (24.0%)	25 (14.4%)	38 (15.0%)	20 (12.1%)	45 (12.8%)	12 (11.9%)	209 (15.7%)
55-64	77 (26.8%)	60 (34.5%)	62 (24.4%)	48 (29.1%)	111 (31.6%)	17 (16.8%)	375 (28.2%)
65-74	83 (28.9%)	68 (39.1%)	93 (36.6%)	52 (31.5%)	118 (33.6%)	38 (37.6%)	452 (33.9%)
>74	58 (20.2%)	21 (12.1%)	61 (24.0%)	45 (27.3%)	77 (21.9%)	34 (33.7%)	296 (22.2%)
Gender (not significant)^c^							
Female	114 (40.1%)	66 (37.9%)	127 (50.0%)	71 (43.0%)	147 (42.6%)	45 (45.0%)	570 (43.1%)
Male	170 (59.9%)	108 (62.1%)	127 (50.0%)	94 (57.0%)	198 (57.4%)	55 (55.0%)	752 (56.9%)
Education (p < 0.001)^c^							
Low	164 (62.8%)	70 (42.9%)	87 (35.4%)	116 (73.4%)	84 (24.7%)	64 (68.1%)	585 (46.4%)
High	97 (37.2%)	93 (57.1%)	159 (64.6%)	42 (26.6%)	256 (75.3%)	30 (31.9%)	677 (53.6%)
Secondary complications							
Stroke (p < 0.05)^c^							
No	263 (91.0%)	168 (94.9%)	228 (89.4%)	152 (92.1%)	337 (95.7%)	95 (95.0%)	1243 (92.9%)
Yes	26 (9.0%)	9 (5.1%)	27 (10.6%)	13 (7.9%)	15 (4.3%)	5 (5.0%)	95 (7.1%)
Heart (p < 0.01)^d^							
No problems	215 (76.0%)	127 (78.4%)	177 (76.6%)	109 (69.0%)	286 (84.4%)	82 (82.8%)	996 (78.3%)
Problems, no attack	40 (14.1%)	20 (12.3%)	32 (13.9%)	28 (17.7%)	30 (8.8%)	8 (8.1%)	158 (12.4%)
Heart attack	28 (9.9%)	15 (9.3%)	22 (9.5%)	21 (13.3%)	23 (6.8%)	9 (9.1%)	118 (9.3%)
Kidney (not significant)^c^							
No kidney failure	276 (99.6%)	173 (100.0%)	243 (99.2%)	158 (99.4%)	348 (100.0%)	100 (100.0%)	1298 (99.7%)
Kidney failure	1 (0.4%)	0 (0.0%)	2 (0.8%)	1 (0.6%)	0 (0.0%)	0 (0.0%)	4 (0.3%)
Lower extremities (p < 0.001)^d^							
No problems	230 (81.3%)	149 (86.1%)	185 (73.4%)	144 (87.8%)	318 (93.8%)	83 (83.8%)	1109 (84.7%)
Moderate problems	43 (15.2%)	23 (13.3%)	63 (25.0%)	18 (11.0%)	21 (6.2%)	15 (15.2%)	183 (14.0%)
Amputations	10 (3.5%)	1 (0.6%)	4 (1.6%)	2 (1.2%)	0 (0.0%)	1 (1.0%)	18 (1.4%)
Eyes (p < 0.001)^d^							
No problems	209 (73.6%)	155 (89.1%)	202 (79.5%)	134 (81.7%)	315 (91.0%)	73 (73.0%)	1088 (82.3%)
Moderate problems	74 (26.1%)	19 (10.9%)	52 (20.5%)	29 (17.7%)	31 (9.0%)	26 (26.0%)	231 (17.5%)
Blind	1 (0.4%)	0 (0.0%)	0 (0.0%)	1 (0.6%)	0 (0.0%)	1 (1.0%)	3 (0.2%)
EQ-5D-3L items							
Mobility (p < 0.001)^d^							
No problems	142 (49.3%)	104 (58.8%)	167 (65.5%)	84 (50.9%)	242 (68.8%)	58 (58.0%)	797 (59.6%)
Some problems	143 (49.7%)	73 (41.2%)	88 (34.5%)	81 (49.1%)	110 (31.3%)	41 (41.0%)	536 (40.1%)
Confined to bed	3 (1.0%)	0 (0.0%)	0 (0.0%)	0 (0.0%)	0 (0.0%)	1 (1.0%)	4 (0.3%)
Self-care (p < 0.001)^d^							
No problems	212 (73.4%)	157 (88.7%)	223 (87.5%)	149 (90.3%)	330 (94.8%)	83 (83.0%)	1154 (86.5%)
Some problems	67 (23.2%)	20 (11.3%)	30 (11.8%)	13 (7.9%)	15 (4.3%)	15 (15.0%)	160 (12.0%)
Unable	10 (3.5%)	0 (0.0%)	2 (0.8%)	3 (1.8%)	3 (0.9%)	2 (2.0%)	20 (1.5%)
Usual activities (p < 0.001)^d^							
No problems	158 (54.9%)	129 (72.9%)	182 (71.7%)	110 (67.1%)	272 (77.3%)	71 (70.3%)	922 (69.0%)
Some problems	103 (35.8%)	45 (25.4%)	67 (26.4%)	47 (28.7%)	77 (21.9%)	25 (24.8%)	364 (27.2%)
Unable	27 (9.4%)	3 (1.7%)	5 (2.0%)	7 (4.3%)	3 (0.9%)	5 (5.0%)	50 (3.7%)
Pain/discomfort (p < 0.001)^d^							
No	120 (41.5%)	77 (44.0%)	76 (30.0%)	65 (39.6%)	200 (56.8%)	43 (42.6%)	581 (43.6%)
Moderate	129 (44.6%)	93 (53.1%)	158 (62.5%)	92 (56.1%)	141 (40.1%)	48 (47.5%)	661 (49.6%)
Extreme	40 (13.8%)	5 (2.9%)	19 (7.5%)	7 (4.3%)	11 (3.1%)	10 (9.9%)	92 (6.9%)
Anxiety/depression (p < 0.001)^d^							
Not	156 (54.9%)	156 (88.6%)	175 (70.3%)	34 (20.9%)	302 (85.6%)	56 (56.0%)	879 (66.3%)
Moderately	108 (38.0%)	17 (9.7%)	67 (26.9%)	97 (59.5%)	50 (14.2%)	40 (40.0%)	379 (28.6%)
Extremely	20 (7.0%)	3 (1.7%)	7 (2.8%)	32 (19.6%)	1 (0.3%)	1 (0.3%)	67 (5.1%)

^a^Due to missing values the numbers of cases evaluated for each variable are mostly lower than the sample sizes. Percentages refer to the ‘within country’ distributions of the variables (columns).

^b^Test for country differences performed with multiple linear regression with ‘country’ as dummy coded independent variable.

^c^Test for country differences performed with Pearson’s chi-square test.

^d^Test for country differences performed with Kruskal-Wallis test.

### Generalizability of findings concerning EQ-5D-3L items

No Spanish participant between 55 and 64 years of age reported problems with self-care and all Spanish participants with a previous stroke reported problems with mobility, usual activities and pain or discomfort (data not shown). Hence, the regression models with interaction terms could not be computed for these combinations of EQ-5D-3L items and test variables (see Table 3). All other originally intended analyses, however, could be performed, i.e. the significance of interactions could be tested in 31 cases.

**Table 3:** **Model fits for data from all six study countries** ^**a**^

	**Mobility**	**Self-care**	**Usual activities**	**Pain/Discomfort**	**Anxiety/Depression**
Age	0.13; 0.15	0.14; n.d.^b^	0.10; 0.12	0.07; 0.09	0.26; 0.27
Gender	0.07; 0.08^*^	0.10; 0.11	0.07; 0.08	0.09; 0.10	0.27; 0.27
Education	0.04; 0.04	0.10; 0.10	0.06; 0.06	0.05; 0.06	0.27; 0.27
Previous stroke	0.07; n.d.^b^	0.13; 0.13	0.11; n.d.^b^	0.05; n.d.^b^	0.27; 0.27
Problems with heart	0.08; 0.09	0.13; 0.14	0.09; 0.09	0.06; 0.07^*^	0.26; 0.26
Problems with lower extremities	0.16; 0.17	0.19; 0.19	0.14; 0.14	0.12; 0.13	0.29; 0.29
Problems with eyes	0.09; 0.09	0.15; 0.16^*^	0.08; 0.09	0.08; 0.08	0.27; 0.28

^a^First number in each cell is Nagelkerke’s R^2^ for the model without interaction terms, the second number is Nagelkerke’s R^2^ for the model with interaction terms. Asterisks behind the second number symbolise the results of the test as to whether the second model explains the dependent variable significantly better than the first. ^*^ = ‘p < 0.05’.

^b^n.d. means not determinable.

In only 3 of the 31 cases in which interactions could be tested, the interaction terms raised the model fit significantly with a significance level of 0.05 (see Table 3). These three analyses concerned the relationships of the item ‘mobility’ with gender (increase in Nagelkerke’s Pseudo R^2^: 0.012, p < 0.05), of the item ‘self-care’ with problems with eyes (increase in Nagelkerke’s Pseudo R^2^: 0.015, p < 0.05), and of the item ‘pain/discomfort’ with problems with heart (increase in Nagelkerke’s Pseudo R^2^: 0.012, p < 0.05). In all three of these cases the regression coefficient for Spain deviated most from the corresponding coefficient for the model computed for all countries without the interaction terms as independent variables. When 31 significance tests with a significance level of 0.05 are performed then the probability that more than two tests yield a significant result by chance is 0.201. Therefore, the three significant results could very well be false rejections of the zero-hypothesis. Of course, with a larger sample size more effects might be detected. However, in those three cases where statistically significant interaction effects were found, the differences between the Nagelkerke’s Pseudo R^2^ are small. Hence, effects detected with a larger sample size can also be expected to be quite small. So, the results presented here suggest that the six investigated different language versions relate in approximately the same way to the test variables. However, the fact that the regression coefficients for Spain deviate most in all three significant interactions might also indicate that either the items of the Spanish version are differently understood by the respondents, or that they actually relate differently to the test variables.

To investigate the generalizability of findings across the five countries other than Spain, the same analyses were repeated without the Spanish data. All 35 interaction effects could be tested and only one of these 35 tests, i.e. the interaction between countries and educational level with regard to the item ‘pain/discomfort’, yielded a statistical significant result on the 0.05 level (see Table 4). When 35 statistical tests with a significance level of 0.05 are performed then the probability that at least one test yields a significant result by chance is 0.834. Therefore, the one and only significant result can very well be a false rejection of the zero-hypothesis. Considering the patterns of Nagelkerke’s pseudo R-square (see Table 4) no large effects can be expected to be detected with a larger sample size. Consequently, the results provide strong evidence for the generalizability of the results across England, Finland, Germany, Greece and the Netherlands.

**Table 4:** **Model fits without Spanish data** ^**a**^

	**Mobility**	**Self-care**	**Usual activities**	**Pain/Discomfort**	**Anxiety/Depression**
Age	0.14; 0.15	0.14; 0.16	0.10; 0.12	0.07; 0.09	0.27; 0.29
Gender	0.07; 0.07	0.11; 0.11	0.07; 0.07	0.08; 0.09	0.28; 0.29
Education	0.04; 0.05	0.10; 0.11	0.06; 0.06	0.06; 0.07^*^	0.28; 0.29
Previous stroke	0.07; 0.07	0.13; 0.14	0.10; 0.11	0.06; 0.06	0.28; 0.28
Problems with heart	0.09; 0.10	0.13; 0.15	0.09; 0.10	0.07; 0.08	0.28; 0.28
Problems with lower extremities	0.15; 0.16	0.19; 0.19	0.14; 0.15	0.12; 0.13	0.30; 0.30
Problems with eyes	0.09; 0.10	0.17; 0.18	0.10; 0.11	0.09; 0.09	0.29; 0.29

^a^First number in each cell is Nagelkerke’s R^2^ for the model without interaction terms, the second number is Nagelkerke’s R^2^ for the model with interaction terms. Asterisks behind the second number symbolise the results of the test as to whether the second model explains the dependent variable significantly better than the first. ^*^ = ‘p < 0.05’.

### Validity of the EQ-5D-3L items

#### Relation between EQ-5D-3L items and test variables

The validity of the EQ-5D-3L items was only investigated with the data of those five countries for which there was strong evidence of generalizability. For these data the results from the regression analyses performed with the test variables and dummy coded countries but without interaction terms as independent variables were inspected more thoroughly (see Table 5). These results show that the first four items of the EQ-5D-3L, i.e. ‘mobility’, ‘self-care’, ‘usual activities’ and ‘pain/discomfort’, relate in the same way to the seven test variables. The tendency of reporting problems increases in a statistically significant manner with age, female gender, previous stroke, heart problems, problems with lower extremities and problems with eyes. The tendency of reporting problems also increases with lower education for all four items, but only for three of those items in a statistically significant manner. The statistical test for the item ‘self-care’ just misses a significant result; but the regression coefficient for this item (b = −0.35) deviates more from zero than those for the items ‘mobility’ (b = −0.33) and ‘pain/discomfort’ (b = −0.33) and the confidence intervals for all four items overlap largely (see Table 5). There are still further commonalities between the first four items. For the three socio-demographic variables the absolute value of the coefficient is always largest for the highest age category in comparison with the lowest age category and always lowest for education. For the four investigated secondary complications this value is always largest for problems with lower extremities.

**Table 5:** **Regression coefficients for models without interaction terms, Spanish data excluded** ^**a**^

	**Mobility**	**Self-care**	**Usual activities**	**Pain/Discomfort**	**Anxiety/Depression**
Age					
<55 (reference)					
55-64	0.45*	0.19	0.24	0.34	0.11
	0.05; 0.85	−0.42; 0.80	−0.18; 0.65	−0.02; 0.69	−0.31; 0.52
65-74	0.72***	0.43	0.32	0.35	0.03
	0.33; 1.11	−0.15; 1.01	−0.08; 0.72	−0.00; 0.69	−0.38; 0.43
>74	1.82***	1.38***	1.30***	0.92***	−0.14
	1.40; 2.25	0.80; 1.96	0.88; 1.73	0.53; 1.31	−0.59; 0.30
Male gender	−0.61***	−0.46**	−0.61***	−0.68***	−0.46**
	−0.85; −0.38	−0.80; −0.11	−0.86; −0.36	−0.91; −0.44	−0.73; −0.18
Higher education	−0.33*	−0.35	−0.36**	−0.33*	−0.03
	−0.59; −0.08	−0.73; 0.03	−0.63; −0.09	−0.59; −0.08	−0.33; 0.27
Previous stroke	1.28***	1.41***	1.72***	0.65**	0.87***
	0.81; 1.75	0.92; 1.90	1.25; 2.20	0.17; 1.14	0.40; 1.35
Problems with heart	0.98***	0.86***	0.89***	0.68***	0.45**
	0.69; 1.27	0.47; 1.24	0.60; 1.18	0.38; 0.98	0.13; 0.77
Problems with lower extremities	1.81***	1.67***	1.62***	1.56***	0.95***
	1.44; 2.18	1.27; 2.06	1.27; 1.96	1.14; 1.99	0.59; 1.31
Problems with eyes	1.17***	1.46***	1.72***	0.99***	0.76***
	0.85; 1.49	1.09; 1.84	1.25; 2.20	0.64; 1.34	0.43; 1.10

^a^First entry in the cell is the regression coefficient, i.e. the logarithm of the odds ratio. The asterisks behind this entry mark the results of the significance test for deviation from zero, with * = ‘p < 0.05’, **=’p < 0.01’, ***=’p < 0.001’. The numbers in the second row of each cell are the boundaries of the 95%-interval of confidence for the regression coefficient.

However, the regression coefficients also indicate a slight difference between the first three items, i.e. ‘mobility’ , ‘self-care’ and ‘usual activities’ , and the item ‘pain/discomfort’. The latter item seems to be less affected by age (b for age > 74: 0.92) and by the four secondary complications of diabetes (b range: 0.65 to 1.56) than the other three items (see Table 5). For ‘age’ and for ‘previous stroke’ this tendency is even expressed in the confidence intervals, i.e. for the age category ‘> 74’ the confidence interval for ‘pain/discomfort’ (0.53; 1.31) does not overlap with the confidence interval for the item ‘mobility’ (1.40; 2.25), and for ‘previous stroke’ the confidence interval for ‘pain/discomfort’ (0.17; 1.14) does not overlap with the confidence interval for the item ‘usual activities’ (1.25; 2.20) (see Table 5). There is still a further difference between the first three EQ-5D-3L items and the item ‘pain/discomfort’. Whereas ‘problems with the heart’ constitute that secondary complication which has the smallest effect on the first three items (b range: 0.86 to 0.98), it is ‘previous stroke’ which has the smallest effect (b = 0.65) on the item ‘pain/discomfort’.

The item ‘anxiety/depression’ differs quite distinctly from the other four EQ-5D-3L items. In contrast to the other items it is virtually unrelated to age and education. Moreover, the regression coefficients indicate that it is less affected by the secondary complications with only ‘previous stroke’ being an exception. For ‘problems with lower extremities’ and for ‘problems with eyes’ this tendency is also evident in the confidence intervals, i.e. for ‘problems with lower extremities’ the confidence interval for ‘anxiety/depression’ (0.59; 1.31) does not overlap with the confidence interval for ‘mobility’ (1.44; 2.18), and for ‘problems with eyes’ the confidence interval for ‘anxiety/depression’ (0.43; 1.10) does not overlap the confidence interval for ‘usual activities’ (1.25; 2.20) (see Table 5).

#### Relationships to previous studies

The finding that the first three EQ-5D-3L items are most similar to each other whereas the last item, i.e. ‘anxiety/depression’ , differs most is very well in line with results from factor analyses performed in other studies [25-27]. The finding that health impairments as reflected by more objective indicators have an adverse effect on all five items is, with a few exceptions [6-8,12,13,16], also in line with the findings in earlier studies [6-17]. As in the results presented here, other studies show that age has an adverse effect on the first four EQ-5D-3L items [6,11,14,17,19,20]. However, in contrast to the findings presented here, age also had a statistically significant adverse effect on the item ‘anxiety/depression’ in most of the studies in which this relation has previously been investigated [11,14,17,19,20]. In only one of these studies was no such effect found [6]. As in the study presented here, women reported more problems than men for all five items in most of the previous studies in which this relationship had been investigated [11,14,17,19,20]. There was only one exception [15]. There is also rich evidence that, just as in the study presented here, the tendency of reporting problems for the first four EQ-5D-3L items increases with lower education [11,14,15,17,19,20]. However, in contrast to the study presented here, previous studies also show a statistically significant positive effect of education on the item ‘anxiety/depression’ [11,14,15,17] or at least a non-significant tendency in this direction [19,20].

#### Integrative interpretation of the results relevant for validity

Considered from only a conceptual perspective the constructs addressed by the five EQ-5D-3L items are related in a different way to each other. The first three items are all concerned with bodily function which to a large degree can be affected by the same impairments; the fourth item, i.e. ‘pain/discomfort’, describes something which is physical but which is not necessarily related to functional restrictions; the fifth item, i.e. ‘anxiety/depression’, describes something which is not physical and which can be affected by quite different factors other than physical health. These conceptual relations between the items are very well reflected by the results found in the study presented here as well as by the results of the previous studies concerning the factorial structure of the items [25-27]. Hence, these results provide evidence for the internal validity of the items.

Both the study presented here and previous studies show that more problems are reported for the first four EQ-5D-3L items with increasing age and lower education. As physical health is known to decrease with age and lower education [28-31] these findings further corroborate these items’ validity. The relationships of age and education with the item ‘anxiety/depression’, however, are more complicated.

For the relationship between age and the item ‘anxiety/depression’ two antagonistic mediating mechanisms can be assumed. One mechanism is that physical health decreases with age and that, this in turn, increases anxiety and/or depression [32-34]. On the other hand, most people at an older age receive a pension income from which they can live and which can alleviate some reasons for being anxious and/or depressive. Moreover, older age for some people is associated with a time for reduced work responsibilities and increased leisure time which can also reduce anxiety and/or depression. When these two antagonistic mechanisms work together no universal adverse effect of age on the item ‘anxiety/depression’ can be expected. In the data presented here, both mechanisms might have neutralised each other whereas in most studies in literature the first mechanism might have been stronger.

For the relationship between education and the item ‘anxiety/depression’ two antagonistic mediating mechanisms can also be postulated. One mechanism is that higher education is associated with better physical health [28-31], and better physical health, in turn, is associated with less anxiety and depression [32-34]. The other mechanism is that higher education is very often associated both with more being expected from these people in their work positions and with their own expectations from life in general being higher, which both increases the risk of becoming anxious and depressive. Assuming these two antagonistic mechanisms implies that no universal adverse effect of education on the item ‘anxiety/depression’ can be expected. In the data presented here, both mechanisms might have neutralised each other whereas in most studies in literature the first mechanism might have been stronger.

Both the study presented here and previous studies show that males report fewer health problems than females. The interpretation of these findings, however, is also complicated. In fact, some empirical studies indicate that males are healthier than females [35-38]. However, the classical male self-concept expects males to be both physically and emotionally strong [39], which reduces the tendency to admit impairments even if they actually exist. So, as far as the gender effect in the answers corresponds to actual differences in health this effect constitutes further evidence for the validity of the items. However, to the extent to which the gender effect is stronger than the differences in actual health, this effect is evidence for a response bias and therefore for a specific restriction of validity. With the analyses presented here the relative influence of both factors cannot be determined and additional research would be required.

## Conclusions

The items of the English, Finnish, German, Greek, Dutch, and Spanish versions of the EQ-5D-3L relate in approximately the same way to age, gender, education, previous stroke, heart problems, problems with lower extremities and problems with eyes. However, for 3 of the 31 performed tests statistically significant deviations from the same pattern were detected and all of these were largely attributable to the Spanish data. These results can still be explained by statistical variation caused by chance. However, they might also suggest that something is different either with the European Spanish language version of the EQ-5D-3L or with the actual relationships between the EQ-5D-3L items and the test variables. Further studies are required for deciding between both possible explanations. After excluding the Spanish data, only one of the 35 performed statistical tests of deviation from the same pattern produced a statistically significant result. This provides strong empirical evidence that the items of the English, the Finnish, the German, the Greek, and the Dutch version of the EQ-5D-3L relate in the same way to the test variables. The aggregated results for English, Finnish, German, Greek and Dutch data provide further evidence for the validity of the EQ-5D-3L items.
